# Analysis of physical fitness tests for police officers in various countries: a scoping review

**DOI:** 10.3389/fphys.2025.1703389

**Published:** 2025-11-18

**Authors:** Zhaohua Zhang, Jian He, Xiaohui Zhang, Liru Zhao, Yujian Deng

**Affiliations:** People’s Public Security University of China, Beijing, China

**Keywords:** law enforcement officer, police, physical competence, physical performance, assessment

## Abstract

Physical fitness tests are an essential part of police selection and retention. The aim of this study was to analyze the physical fitness tests of police officers in various countries. We systematically searched Chinese and English databases, including Web of Science, SPORTDiscus, PubMed, and CNKI, from their establishment dates to 1 December 2024. A total of 16 physical fitness tests for police officers from 7 countries were analyzed. All physical fitness tests were divided into two categories: one focused on basic fitness test items (n = 9), and the other was an obstacle courses, the main content of which simulates specific occupational tasks that police officers may need to perform on duty (n = 7). The most used components were cardiorespiratory endurance, muscular strength and endurance and agility. This review includes two distinct testing paradigms: 'General Physical Fitness’ and 'Job-Specific Competence’. It indicates that there is no universally 'optimal’ testing model. The selection or development of police physical fitness tests must be grounded in selection efficiency, ecological validity, and cultural applicability.

## Introduction

Police officers are the core force in maintaining social order and ensuring public security (Marins et al., 2019). The police profession requires them not only to possess legal knowledge and law enforcement skills but also to maintain a good physical condition to cope with the complex and dynamic police duties (Lockie et al., 2019; Rezende et al., 2022). In the course of law enforcement, police officers often encounter complex situations such as physical violence, conflicts, and mass incidents. These situations may require them to perform various physical tasks such as carrying external loads, chasing and arresting suspects, climbing over obstacles, and dragging heavy objects (Tomes et al., 2017). The police profession is characterized by high intensity, high pressure, and high risk. Therefore, police officers need excellent cardiorespiratory endurance, muscular strength and endurance, speed, and agility to perform their duties better and more effectively (Myers et al., 2019; Massuça et al., 2022; Massuça et al., 2023b). Good physical fitness is not only a prerequisite for meeting the demands of police work but also a crucial foundation for enhancing law enforcement effectiveness and ensuring occupational safety (Thielgen and Schade, 2024). Therefore, in order to recruit and train police officers in good condition, it is necessary to optimize the physical fitness tests to suit the current law-enforcement situation.

To date, many countries have already established their own assessment tools to assess police officers’ ability to perform their duties. For example, the Physical Abilities Requirement Evaluation (PARE) and the Police Officer Physical Ability Test (POPAT) were used in different provinces of Canada (Bonneau and Brown, 1995). New Zealand worked with Physical Competence Test (PCT) (Strating et al., 2010; Koedijk et al., 2020). A battery of tests for police officers developed by the Cooper Institute of Aerobic Fitness was used in the United States (The Cooper Institute, 2006; Dawes et al., 2016). However, the methods and contents of physical fitness tests for police officers vary among countries. Therefore, the aim of this scoping review was to review existing police physical fitness tests of various countries and analyze the evaluation methods, contents, application scenarios, etc., of these tools, aiming to provide theoretical references and practical guidance for optimizing and improving the physical fitness tests for police officers in China.

## Methods

This scoping review was performed and reported in accordance with Preferred Reporting Items for Systematic Reviews and Meta-Analysis Extension for Scoping Reviews (PRISMA-ScR) guidelines (Tricco et al., 2018).

### Search strategy

We performed electronic searches in the following four databases: Web of Science Core Collection, SPORTDiscus (EBSCO), PubMed, and China National Knowledge Infrastructure (CNKI) from the establishment of each database to 1 December 2024. The following keywords were used to search for the articles reporting the physical fitness of police: (“physical fitness” OR “physical ability”) AND “police”. Taking Web of Science Core Collection as an example, the search strategy was as follows: #1 TS = (“physical fitness”) OR TS = (“physical ability”); #2 TS = (“police”); #3 #1 AND #2. Additionally, we tracked references from included studies and searched official government websites to expand the scope of literature and materials.

### Inclusion and exclusion criteria

For the study to be included in the final analysis, it had to meet the following criteria: (a) the participants were police officers or probationary police officers; (b) the study focused on the development or application of officially recognized national police physical fitness tests; (c) the study was published in English or Chinese. Exclusion criteria involved: (a) police physical fitness tests not uniformly applied at the national or regional level; (b) the study only reported one or several test items of the police physical fitness tests; (c) the study was published as conference proceedings or reviews; (d) the study has unavailable full texts.

### Study selection and data extraction

After completing the literature search, we used EndNote (version 20.2.1, Clarivate Analytics, Philadelphia, PA, USA) to remove duplicate references. Two researchers used the above-mentioned criteria to review the title and the abstract of each study independently. Then, the full texts of the potentially suitable studies were examined by the same researchers for eligibility. Once the discrepancies regarding the inclusion of a study in the scoping review arose between two researchers, a third researcher was consulted for a final decision.

Data were extracted from the eligible studies and materials using the data extraction table developed by the authors. We extracted the following information: authors and publication year, and details of physical fitness tests (including their name, country of implementation, test items, and application context, etc.).

## Results

### Search results

**FIGURE 1:**
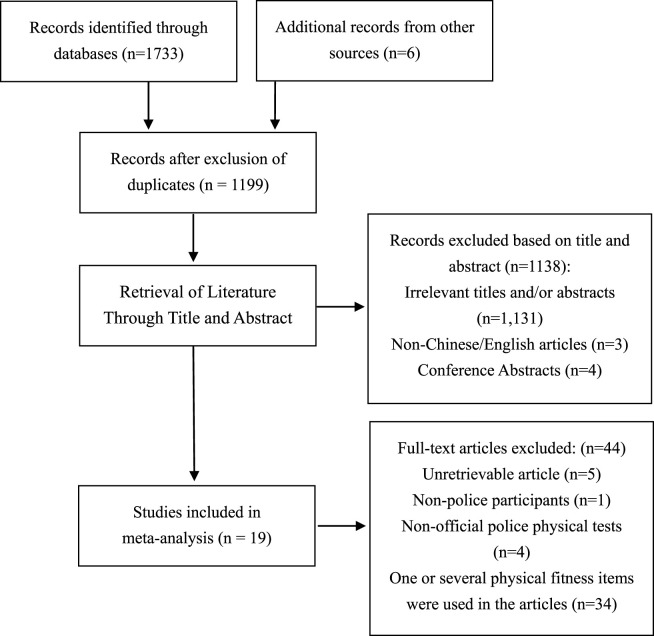
Flow diagram for study inclusion of the scoping review.

The search process is detailed in Figure 1. First, a total of 1,739 records from four databases and official websites were found. After removing duplicates and screening the titles and abstracts, 63 studies remained for full-text assessment. We then retrieved and assessed their full texts; only 19 studies meeting all the inclusion criteria were included in this scoping review.

### Characteristics of police physical fitness tests

A total of 16 police physical fitness tests from seven countries (China, the United Kingdom, South Korea, the Netherlands, Australia, Canada, and the United States) were found. All physical fitness tests can be divided into two categories. One focused on basic fitness test items (n = 9), implemented in countries/regions including China (Mainland, Hong Kong and Macao), South Korea, the United Kingdom, the United States (the Cooper Institute, Michigan, and the New England). The other was obstacle courses simulating specific occupational tasks for police officers (n = 7), utilized in countries such as Australia, the Netherlands, Canada (British Columbia, Alberta, Quebec), the United States (Miami, and New England). In addition, all physical fitness tests can be classified into three categories based on objectives: police recruitment physical fitness tests (n = 14), police promotion physical fitness test (n = 1), and annual physical fitness tests for police officers (n = 3). Notably, the police recruitment physical fitness tests in the Netherlands and South Korea were also used to assess police officers’ physical fitness. Table 1 summarizes the characteristics of police officers’ physical fitness tests.

**TABLE 1:** The description of police physical fitness tests.

Author, year	Country/Region	Name	Assessment items	Application objectives	URL
Song Q L et al. (2022)	China (Mainland)	Physical assessment items and standards for recruitment of people’s police by public security organs	10 m × 4 Shuttle Run 1,000 m/800 m Run Vertical Jump	Police recruitment	https://www.mps.gov.cn/n6557558/c9491275/content.html
Hong Kong Police Force (2025)	China (Hong Kong)	Pre-employment physical fitness test	10 m × 4 Shuttle Run push-ups Vertical Jump 800 m run	Police recruitment	https://www.police.gov.hk/ppp_sc/15_recruit/fitness.html
Macau Special Administrative Region Office of the Secretary for Security (2025)	China (Macao)	Physical fitness test	80 m sprint Cooper Test Sit-ups Pull-ups (male)/Push-ups (female) Long jump High jump Balance beam test	Police recruitment	https://bo.io.gov.mo/bo/i/2022/22/despseg_cn.asp#60
Macau Special Administrative Region Office of the Secretary for Security	China (Macao)	Physical fitness test	Wall vault (male) High jump Long jump (male) Elevated balance beam test Pull-ups (for males under 39) Push-ups (for females and males over 39) Sit-ups 80 m Sprint Cooper test (12-min run)	Promotion	https://bo.io.gov.mo/bo/i/2022/22/despseg_cn.asp#60
Kent Police	The United Kingdom (England, Wales)	Job related fitness test (JRFT)	The 15 m multi-stage fitness test (15 m MSFT) or Chester treadmill police walking and running test (CTPWT and CTPRT) for officers with lower limb injuries	Police recruitment	https://www.kent.police.uk/foi-ai/kent-police/Policy/operational-partnerships/job-related-fitness-test-sop-o43l/
Kim and Kim (2019), Kim et al. (2020)	Korea	Physical performance test	100 m sprint Push-ups Sit-ups Long jump Sit-and-reach test Long-distance running (1.5 km and 3 km)	Police recruitment and annual assessment	https://www.police.go.kr/index.do
South Australia Police Safer Communities (2025)	South Australia	Physical and agility testing	Complete the following tests with a 10 kg vest 80 m sprint Run 25 m carrying two 15 kg weights Cross a slippery balance beam Do 20 step-ups Climb over a 1-m-tall fence Drop to the ground and stand up five times Collect a training firearm and pull the trigger 13 times with each hand	Police recruitment	https://www.police.sa.gov.au/join-us/achievemore/police-officer-careers/recruitment-process
Strating et al. (2010), Koedijk et al. (2020)	Dutch	Physical competence test (PCT)	Obstacle course (A total run of 226.5 m) Climb an obstacle (1.1 m) and jump over low obstacles Push a 200 kg cart three times over a distance of 6 m Pull the cart two times over the same distance Lift and carry a ball of 5 kg 18 times for 3 m a time Drag a 48 kg dummy casualty for 5 m	Police recruitment and annual assessment	
Bonneau and Brown (1995)	Canada (British Columbia)	Police officer physical ability test (POPAT)	(1) Obstacle course Jump over a 6-foot mat Run stairs Jump over the 18-inch hurdles (2) Push/Pull assessment Push/pull a 50-lb weight (6 each) (3) Modified squat-thrust-barrier and vault rail Jump over a 3-foot vault rail (4) Torso bag carry Carry a 100-lb weight over 50 feet	Police recruitment	https://bluelinefitnesstesting.com/popat-alberta/
Bonneau and Brown (1995)	Canada (Alberta/Royal Canadian Mounted Police)	Physical abilities requirement evaluation (PARE)	(1) Obstacle CourseJump over a 5-foot mat Run stairs Jump over two 18-inch hurdles Vault over a 3-ft high railing (2) Push/Pull Push/pull a 70-lb weight (6 each) Controlled falls (front and back falls (2 each)) (3) Torso bag carry Carry a 100-lb weight for 50 feet	Police recruitment	https://bluelinefitnesstesting.com/pare-testing-alberta/
Poirier et al. (2022)	Canada (Quebec)	Physical ability test (PAT)	Complete the following tests with a 6.8 kg weight Move culvert (75 H × 106 W x 103L cm)​ Cross 89.5 cm barrier Step through window (97.5 H × 136 W cm) Slaloms through 6 silhouettes Run stairs Ascend and descend a flight of 6 stairs Climbs over a 183-cm wall Jump over a 122-cm-long obstacle Steps over a 105-cm-high barrier Crawl through a tunnel (65 H × 115 W x 370L cm)​ Climb over a 185-cm chain-link fence Jump over a 61-cm trash can Walk on a barrier (25W x 365L cm)Grab a flashlight and target aiming from behind barrier	Police recruitment	
The Cooper Institute (2006), Dawes et al. (2016)	The United States	Physical fitness test	1.5-Mile run 300 m run Vertical jump 1 RM bench press 1- minute push-ups 1- minute sit-ups 1 RM leg press Sit-and-reach test	Police recruitment	
Crawley et al. (2016)	The United States (Michigan)	Michigan commission on law enforcement standards law enforcement fitness testing	Push-ups Sit-ups Vertical jump 1/2-mile shuttle run	Police recruitment	https://www.michigan.gov/mcoles/standard-training/testing/pre-enrollment-physical-fitness-test
Orr R. M. et al. (2021), Lockie et al. (2023)	New England	Physical competence test (PCT)	2.4 km run Vertical jump Push-ups Grip strength	Police recruitment	https://www.newcops.govt.nz/can-i-be-a-cop/recruitment-process/pat
Dawes et al. (2022), Lockie et al. (2023)	New England	Physical competence test (PCT)	400 m obstacles course Push a 450 kg trailer 10 m Remove a wheel (22 kg) from the trailer and carry 10 m 200 m run Traverse a 5 m “L” beam (1 m high) 1.8 m long jump Vault 1 m high obstacle 30 m agility run Climb window (1 m) and vault wall (1.8 m) Run backwards for 7.5 m with 75 kg weight Climb a 2.2 m high wire fence	Biennial assessment	
Lockie et al. (2024)	The United States (Miami)	Job-related task assessment (JTA)	Obstacles course Ran approximately 370.33 m in an anti-clockwise direction Climb over a 1.22 m wall Crawl under and through an enclosure (0.91 m × 1.52 m) Weave through five cones Climb up and over a 3.66 m ladder structure Drag a 68.04 kg dummy over 15.24 m Run a 5.49 m balance beam and change direction every 1.83 m Climb through a window Run stairs climb three times Push and pull an 81.65 kg sled over 4.57 m	Police recruitment	https://www.miamidade.gov/mdpsti/library/jta-documents.pdf

### Description of police physical fitness tests

All nine police physical fitness tests, which focus on basic fitness, include cardiorespiratory endurance test items. Cardiorespiratory endurance measurement methods used in different tools vary, such as 800 m/1,000 m runs, 12-min run tests, 15 m shuttle runs, 1.5-mile runs, 1/2-mile shuttle runs, and 2.4 km runs. Five of the police fitness tests include lower-body power tests measured by vertical jump. For muscular strength and endurance assessments, Physical Assessment Items and Standards for Recruitment of People’s Police by Public Security Organs in China only measure the lower-body power, while the other eight police physical fitness tests evaluate muscular endurance through push-ups and sit-ups—the most common methods used. The 4 × 10-m shuttle run, used to assess agility and speed ability in police applicants, is adopted in Mainland China and Hong Kong.

All seven police physical fitness tests are obstacle courses, which evaluate an police officer’s ability to perform occupation-specific tasks. They primarily simulate scenarios often encountered in police duty that include foot chase, physical control, obstacle crossing, and carrying to safety. The PARE test is an occupational test that requires police officers to be in good physical shape, have a fit cardiovascular system, as well as good muscular strength and endurance. The PARE test includes eight subtasks in total to simulate a critical incident of chasing, controlling, and apprehending a suspect. The POPAT is designed to evaluate whether candidates meet the minimum physical requirements for performing essential police duties. The POPAT test consists of four sections: obstacle run, push/pull station, modified Squat-Thrust-and-Stand (STAS) activity and weight carry four sections, which are similar to the PARE test. The Dutch National Police used the Physical Competence Test (PCT), based on the PARE test, to measure job-specific fitness (Koedijk et al., 2020). In addition, the following police fitness tests, such as the physical ability test (PAT) in Quebec of Canada, the physical competence test in New England (PCT), the physical and agility testing (PAT) in South Australia, and the job-related task assessment (JAT), have similar designs to the PARE and POPAT tests, which require completing the obstacle courses within the specified time. The main purpose of these tests is to assess the cardiovascular endurance and muscular strength and endurance required for police officers to perform their operational duties.

## Discussion

This scoping review is the first to systematically summarize standardized physical fitness test tools for police officers across nations and regions worldwide. From a global perspective, this study revealed a consensus on the importance of cardiorespiratory endurance and muscular strength and endurance in police physical fitness tests, which were consistent with previous studies (Marins et al., 2019; Massuça et al., 2022; Rasteiro et al., 2023). The reasons may be that cardiorespiratory endurance, muscular strength and endurance are not only related to the health status of police officers, but also crucial factors determining police occupational performance and injury risks (Lindsay et al., 2021). For example, foot chase and carrying to safety are closely related to the level of cardiorespiratory endurance. Muscular strength and endurance play a vital role in completing police tasks such as physical control, dragging, crossing obstacles and climbing over walls (Beck et al., 2015; Marins et al., 2019; Orr R. et al., 2021). Empirical studies also showed that police officers with high levels of cardiorespiratory endurance, muscular strength and endurance had a lower risk of injury in their occupations (Crawley et al., 2016).

Notwithstanding this consensus, the study found significant divergences in the conceptual frameworks and operational methodologies of these tests. Some countries or regions, such as China, South Korea, New England, employ a universal physical fitness test that is identical to that administered to the general population. In contrast, others utilize a job-specific test. The two police physical fitness test models are related (Beck et al., 2015; Lockie et al., 2018; Lockie et al., 2023), but notable differences exist between them. The strengths of the former (e.g., 1.5-Mile run, push-ups) include high standardization, ease of administration, low cost, and the capacity to easily establish norms. Therefore, they are applicable for the initial screening of a large number of candidates during the police recruitment phase, in order to identify future police officers who meet the physical fitness requirements. A key limitation is inadequate ecological validity, as the test items may poorly simulate actual police tasks (e.g., chasing suspects, overcoming obstacles). This may result in overlooking candidates with good fitness who possess superior situation-specific physical capacities. Conversely, the obstacle course test demonstrates high face and ecological validity, as it directly simulates law enforcement scenarios (Koedijk et al., 2020; Lockie et al., 2024). It assesses not only physical fitness but also comprehensively evaluates decision-making, coordination, and skill application under pressure. Its drawbacks include not only logistical challenges like complex facilities and equipment, high rater demands, and a greater injury risk, but also performance being more influenced by specific skill training than by foundational fitness. The core issue in police selection and assessment is whether to measure 'universal foundational fitness’ or 'job-specific task competence’. In the future, the research could explore the development of an integrated model that utilizes general physical fitness tests for efficient initial screening during recruitment, while incorporating job-specific tests for in-depth assessment during promotions or specialized unit selections. A key strength of this study is its extensive coverage and systematic analysis, offering the first global mapping of official police fitness tests. This work establishes a foundation for future cross-national and cross-cultural comparative research. In addition, there are several limitations in this study. First, due to the strict inclusion criteria, some standardized police physical fitness tests that are not used at the national or regional level may have been overlooked, such as the physical fitness battery used by the Portuguese police academy to assess the fitness of police cadets (Kim and Kim, 2019; Massuça et al., 2023a). Second, reporting bias is also a more significant limitation. We analyzed officially released testing documents, but we were unable to ascertain the fidelity with which these police physical fitness tests are implemented in practice or any undocumented informal modifications. Third, this study primarily focused on what is tested and how it is tested, but provided limited exploration of the policy-making processes, historical evolution, and cultural contexts behind why the tests are designed as they are. These underlying drivers are crucial for understanding the variations in testing approaches and represent an important direction for future qualitative research. In the future, it is imperative to conduct large-scale empirical studies that directly compare the predictive validity of general fitness tests and occupation-specific tests regarding real-world job performance, injury rates, and long-term occupational health. In addition, it is necessary to explore how to establish a more scientific and standardized scoring system for the obstacle course in future research.

## Conclusion

A total of 16 police physical fitness tests were included in this study. There is a consensus that cardiorespiratory endurance, muscular endurance and strength are fundamental to police competency. However, it includes two distinct testing paradigms: 'general physical fitness’ and 'job-specific task competence’. This review indicates that there is no universally 'optimal’ testing model. The selection or development of police physical fitness tests must be grounded in selection efficiency, ecological validity, and cultural applicability.
